# Sensitivity Analysis of Single- and Bimetallic Surface Plasmon Resonance Biosensors

**DOI:** 10.3390/s21134348

**Published:** 2021-06-25

**Authors:** Piotr Mrozek, Ewa Gorodkiewicz, Paweł Falkowski, Bogusław Hościło

**Affiliations:** 1 Faculty of Mechanical Engineering, Bialystok University of Technology, Wiejska 45C, 15-351 Bialystok, Poland; b.hoscilo@pb.edu.pl; 2 Bioanalysis Laboratory, Faculty of Chemistry, University of Bialystok, Ciolkowskiego 1K, 15-245 Bialystok, Poland; ewka@uwb.edu.pl (E.G.); pawelfalkowski@wp.pl (P.F.)

**Keywords:** surface plasmon resonance, bimetallic biosensor, sensitivity, cathepsin determination, biomarkers

## Abstract

Comparative analysis of the sensitivity of two surface plasmon resonance (SPR) biosensors was conducted on a single-metallic Au sensor and bimetallic Ag–Au sensor, using a cathepsin S sensor as an example. Numerically modeled resonance curves of Au and Ag–Au layers, with parameters verified by the results of experimental reflectance measurement of real-life systems, were used for the analysis of these sensors. Mutual relationships were determined between ∂Y/∂n components of sensitivity of the Y signal in the SPR measurement to change the refractive index n of the near-surface sensing layer and ∂n/∂c sensitivity of refractive index n to change the analyte’s concentration, c, for both types of sensors. Obtained results were related to experimentally determined calibration curves of both sensors. A characteristic feature arising from the comparison of calibration curves is the similar level of Au and Ag–Au biosensors’ sensitivity in the linear range, where the signal of the AgAu sensor is at a level several times greater. It was shown that the influence of sensing surface morphology on the ∂n/∂c sensitivity component had to be incorporated to explain the features of calibration curves of sensors. The shape of the sensory surface relief was proposed to increase the sensor sensitivity at low analyte concentrations.

## 1. Introduction

Surface plasmon resonance (SPR) is an optical measuring technique applied to test the refractive index changes occurring in very close proximity to a thin, metallic layer in which collective resonant oscillations of free electrons have been induced [1,2]. The foundation of SPR biosensors’ design is a metal–dielectric interface, the surface of which is functionalized by immobilization of the bioreceptor or selective ligand with respect to the analyte being detected. The interaction between the immobilized bioreceptor and captured analyte particles modifies SPR conditions, which, in turn, generate a change of the sensor’s reflectance, which can be measured and correlated with the change of the analyte’s concentration. SPR biosensors have found applications in many fields, including in environmental protection [3,4,5,6,7,8], biological testing [9,10,11,12,13,14], food safety [15,16,17,18,19,20], and clinical diagnostics [21,22,23,24,25,26]. 

The sensitivity of measurement is one of the most important functional features of SPR biosensors [2,27,28]. Sensitivity S expresses the ratio of change of the sensor’s output signal Y value and the change of the measured value X, and is equal to the value of the calibration curve’s slope:

(1) $$S=\frac{\partial Y}{\partial X}$$

In the case of an SPRi biosensor with modulation of intensity i as a function of analyte concentration c, sensitivity S_ic_ can be expressed as:

(2) $$S_{ic}=\frac{\partial Y}{\partial c}=\frac{\partial Y}{\partial n}\frac{\partial n}{\partial c}=S_{RI}S_{nc}$$

where n is the refractive index of the dielectric medium at the sensing surface, S_RI_ is the sensitivity of output signal Y to change of the refractive index n caused by bonding of the analyte to the sensor’s biorecognition layer, and S_nc_ the sensitivity of refractive index n to change of analyte concentration c.

In the general case, a single, thin layer of gold, which is used in the majority of SPR biosensors currently applied, is not optimal in terms of the sensitivity requirements posed towards biosensors. In recent years, there have been many publications proposing diverse multi-layer and multi-material biosensor design configurations developed for the purposes of enhancing their sensitivity [29,30,31,32]. Nevertheless, silver and gold are two metallic elements that are used in most modern commercial applications due, accordingly, to their high sensitivity and chemical stability [30,33]. Bimetallic Ag–Au sensors are characterized by the combined advantages of both metals. Bimetallic structures using silver and other metals also proved to be useful in other aspects, apart from increasing the sensitivity of sensory surfaces. In bimetallic Ag and titanium nanoislands on top of SiO_2_ surfaces, Ti proved to suppress the Ag from its oxidation in wet environments of SPR structures [34]. A thin Cu seed layer was shown as an effective approach to produce atomically smooth Ag films with greater electrical conductivity [35]. It was demonstrated that the SPR peaks of Ag–Au bimetallic nanoparticles were tunable over a broad range in the visible region, and this feature could be harnessed to create some attractive optical properties and functionalities that are difficult to achieve with a single-component Ag or Au nanoparticle [36].

Self-assembled monolayers (SAM) as a method of formulation of ultra-thin organic films on Au sensory surface requires fewer steps than other approaches [2]. The SAM of thiolate compounds has been widely used utilizing EDC/NHS chemistry for the coupling of biomolecules [37,38,39]. In this article, cysteamine was used to form SAM film tightly attached to Au surface by use of a mercapto group. The amino groups of cysteamine SAM made it possible to immobilize antibodies using their carboxyl groups activated in EDC/NHS coupling procedure. As a result, a strong covalent–amide bond was formed. Subsequent interaction with analyte molecules was enabled in this way for the sensor operation.

In this work, a comparative analysis of the sensitivity of two SPR biosensors was conducted on a single-metallic Au sensor and bimetallic Ag–Au sensor, using a cathepsin S sensor as an example. In recent years, cathepsin S has emerged as an attractive target for inhibiting immune responses [40]. The continuous presentation of antigenic self peptides is thought to perpetuate the autoimmune disease process. Inhibitors of cathepsin S block the presentation of autoantigens and may hold great promise for novel immunosuppressive therapy. Cathepsin S SPR biosensor may prove useful in this context. In this article, attention was directed to the details of sensing surface morphology that affected the sensor’s sensitivity. As far as the authors know, this is the first proposal in the literature of a description of biosensors’ calibration curves quantitatively relating to sensing surface morphology parameters.

## 2. Materials and Methods

### 2.1. Deposition of Thin, Metallic Ag–Au Layers 

Glass substrates with dimensions 20 × 20 × 1 mm and refractive index n = 1.51, cut out from microscope slides (Thermo Scientific), were first polished using an aqueous suspension of cerium oxide, then cleaned with the use of cleaning agents, i.e., detergent, acetone, and isopropyl alcohol. Between every use of the cleaning agent, slides were rinsed and washed ultrasonically in deionized water. Thin metallic layers were deposited onto the surface of the glass by means of vapor deposition in an NA501 vacuum system in a vacuum of 8 × 10^−6^ ÷ 1 × 10^−5^ hPa. Prior to deposition of the Ag layer, approx. 1.0 nm of adhesive Cr layer (99.9%) was deposited at a rate of approx. 0.1 nm/s. Next, approx. 43 nm of Ag (99.99%) was deposited at a rate of 0.08 nm/s, and approx. 4 nm of Au (99.99%) at a rate of 0.01 nm/s. Layer thickness and deposition rate were monitored by means of a quartz crystal microbalance. Glass substrates, with dimensions of 16 × 16 × 1 mm, with approx. 3 nm of Ti and a single Au layer approx. 50 nm thick were purchased from Ssens (www.ssens.nl).

### 2.2. Materials and Reagents for Biosensing Surface Preparation and Measurements

Cathepsin S protein as well as a rat monoclonal antibody specific for cathepsin S (R&D Systems, USA), cysteamine hydrochloride, N-Ethyl-N′-(3-dimethylaminopropyl) carbodiimide (EDC), human albumin (all SIGMA, Steinheim, Germany), N-hydroxysuccinimide (NHS) (ALDRICH, Munich, Germany) were used, as well as absolute ethanol (POCh, Gliwice, Poland), HBS-ES solution pH = 7.4 (0.01 M HEPES, 0.15 M sodium chloride, 0.005% Tween 20, 3 mM EDTA), phosphate-buffered saline (PBS) pH = 7.4, carbonate buffer pH = 8.50–9.86 (all BIOMED, Lublin, Poland). Aqueous solutions were prepared with miliQ water (Simplicity® MILLIPORE) and alcohol solutions with 99.8% absolute ethyl alcohol (POCh, Gliwice, Poland). All measurements were conducted using glass plates coated with the Au and Ag–Au metal layers described above.

Metallic surfaces of the Au and Ag–Au chips were covered with photopolymer and hydrophobic paint as described in previous papers [41]. Each chip had 9 places with 12 free metallic surfaces of 0.125 mm^2^ each.

### 2.3. Receptor (Antibody) Immobilization 

Glass plates coated with pure metal were first rinsed with ethanol and miliQ and dried under a stream of argon. A monolayer of cysteamine linker was achieved by immersing each metal-coated glass slide in a 20 mM alcohol cysteamine solution for at least 12 h at room temperature. In the second step, the slide was washed with absolute ethyl alcohol and miliQ water and finally, dried under an argon atmosphere. The antibody activation process was driven by carboxyl groups present in the antibody reacting with EDC (250 nM) and NHS (250 nM), in the presence of carbonate buffer (pH = 8.5). Antibody immobilization was conducted by mixing NHS and EDC in a ratio of 1:1 in a solution of carbonate buffer at pH 8.5, after which the antibody solution was added. After activation, the mixture of immobilized antibodies was applied onto the active area of a slide and incubated for 1 hour at 37 °C. After this time, the slide was rinsed 10 times with miliQ water and dried under an argon atmosphere. The sensor prepared in this way was then applied for determination of cathepsin S.

### 2.4. Instrumental Measurements and Numerical Simulations

**Figure 1:**
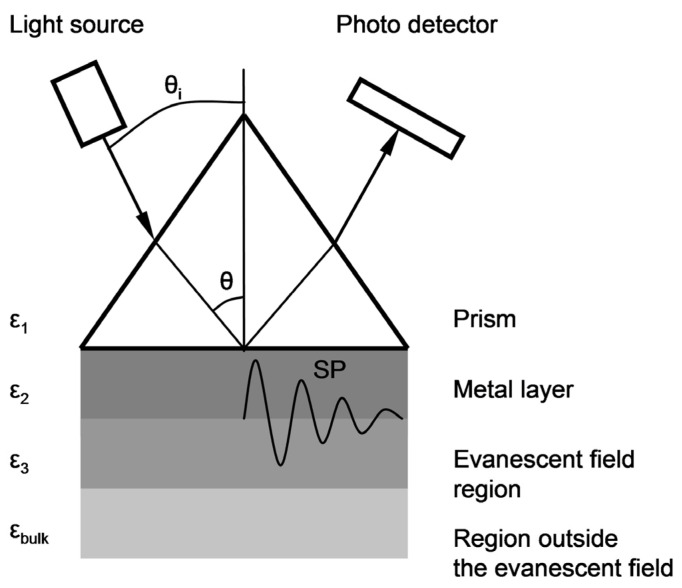
A schematic representation of the basic Kretschmann configuration of prism coupling for reflectivity measurements to determine SPR characteristics.

The surface topography of layers deposited on the glass substrate was measured by atomic force microscopy (AFM) using a Nanosurf Nanite B system in contact mode. Photographs of the surface and fracture surfaces of thin layers were taken using FIB-SEM Scios 2 DualBeam system. SPR characterization of thin films was performed by using a homemade experimental setup assembled in the Kretschmann prism configuration. The prism/sample combination was placed on a revolving table mounted on the shaft of an IP58 programmable incremental encoder with a programmed resolution of 0.01° (Lika Electronics, 0.005° measurement accuracy), driven by a microprocessor-controlled unit. Surface plasmon excitation was achieved by directing a 5 mW P-polarized parallel light beam of a laser diode (wavelength λ = 650 nm) onto the prism/sample interface and measuring the intensity variation of the reflected light as a function of incident angle θ_i_ (Figure 1).

Winspall software (RES-TEC Resonant Technologies GmbH) was used to simulate SPR curves and determine theoretical values of parameters describing optical layers on the basis of fitting model resonance curves to the results determined experimentally with the measuring system in the Kretschmann configuration [42]. 

### 2.5. SPRi Measurements 

SPRi measurements of cathepsin S were performed using a homemade device described in previous papers [41]. In the first step, the proper SPR angle was selected, at which the strongest light contrast between the sensor’s active spaces and its background was achieved. The basic concept of all SPRi measurements involved the recording of a proper image in two different polarization modes: P (light) and S (dark). Since the analytical signal responds to the number of cathepsin S biomolecules bonded at the sensor surface, both types of images were recorded: before and after interacting with the analyte.

Cathepsin S standard solutions were placed directly on the prepared biosensors for 10 min to allow for interaction with the receptor-antibody. The volume of the sample applied to each measuring area was 3 μL. After this time, the biosensor was washed with HBS-ES buffer and water to remove unbound molecules from the surface. SPRi measurements were performed at a constant light angle. Two images were recorded: the first shows the immobilization of the antibody, and the second shows the interaction of the antibody-receptor with the analyte cathepsin S. The intensity of the signals received was obtained after subtracting the background signal of the S polarization. Non-specific binding was monitored by measuring the SPRi signal in the area on the slide without the receptor (ligand). Non-specific binding was minimized by preparing samples in a PBS buffer and by applying BSA in PBS buffer to the slide. The SPRi signal, which was proportional to the mass of entrapped cathepsin S, was obtained as the difference between the signals before and after interacting with the analyte, separately for each area.

Each chip was used for measurements only once. A large number of measurement fields allowed for statistical processing of the results of measurements carried out with the use of one sample. It was not necessary to perform the sensor regeneration phase and the re-measurement phase, typical of the SPR sensors operating under flow conditions, requiring high reproducibility and stability. In order to ensure the invariable parameters of the metallic measurement fields of the sensors, before use, the sensor chips were stored in containers filled with argon. More experimental data will be presented in the next articles on bimetallic chips.

## 3. Results

**Figure 2:**
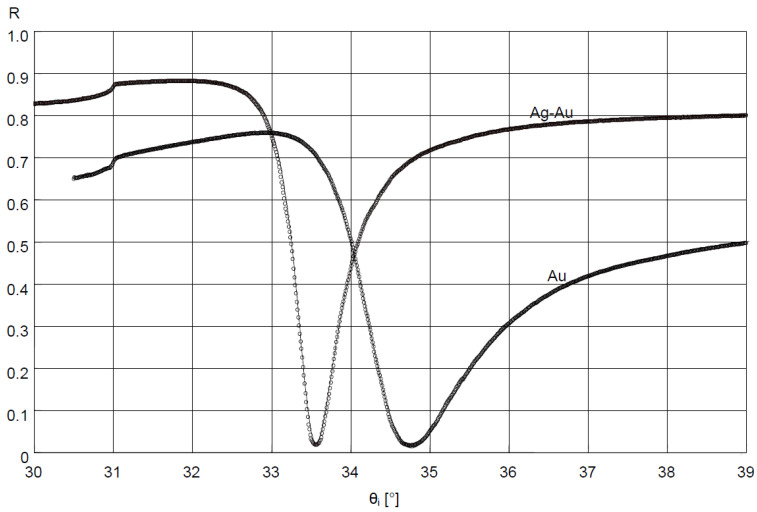
Results of fitting the SPR model curves using Winspall application (solid lines) to the reflectance R measurement results (circles) of deposited Ag–Au and Au layer.

In the measuring system in the Kretschmann configuration, measurements of reflectance R as a function of angle θ_i_ (Figure 1), at a resolution of 0.01°, were conducted for the slides with Au and Ag–Au layers. The results are shown in Figure 2. 

Values of dielectric constants of Cr, Ag, and Au layers were determined for the bimetallic Ag–Au sensor, with the fitting of the model SPR curve to the results of measurement of this layer’s reflectance using the Winspall application (Figure 2). Values of dielectric constants of Ti and Au layers were determined for the single metallic Au sensor in a similar way. For the bimetallic sensor, very good fit of modeling and experimental results was obtained for a thickness of 0.6 nm, 43.0 nm and 3.1 nm, respectively, of modeled Cr, Ag, and Au layers. The values of real and imaginary components of relative electrical permittivity, in fitting of results, amounted to, for Cr, Ag, and Au, respectively: ε_Cr_ = ε_Cr_′+ iε_Cr_″ = −2.1 + i20.9, ε_Ag_ = ε_Ag_′ + iε_Ag_″ = −19.4 + i1.2 and ε_Au_ = ε_Au_′+ iε_Au_″ = −12.8 + i1.4. For the Au sensor, a thickness of 2.9 nm and 47.6 nm, ε_Ti_ = ε_Ti_′+iε_Ti_″ = −4.1 + i17.9 and ε_Au_ = ε_Au_′ + iε_Au_″ = −12.8 + i1.3 for Ti and Au, respectively, was obtained.

**Figure 3:**
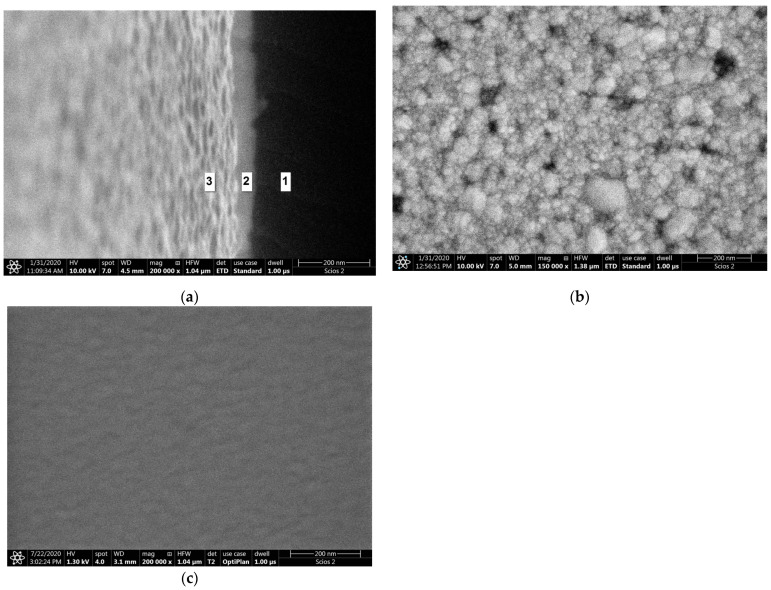
SEM images: (**a**) Ag–Au bimetallic layer surface morphology; (**b**) Ag–Au bimetallic layer in cross-sectional view made for a slightly tilted sample: 1: cross-section of glass substrate, 2: cross-section of Ag–Au metallic layer, 3: view of a tilted Ag–Au top surface; (**c**) Au single-metallic layer surface morphology.

**Figure 4:**
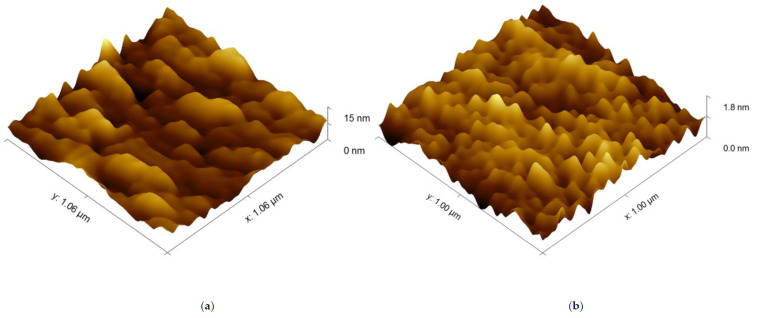
AFM surface topography images: (**a**) Ag–Au bimetallic layer; (**b**) Au single-metallic layer.

**Figure 5:**
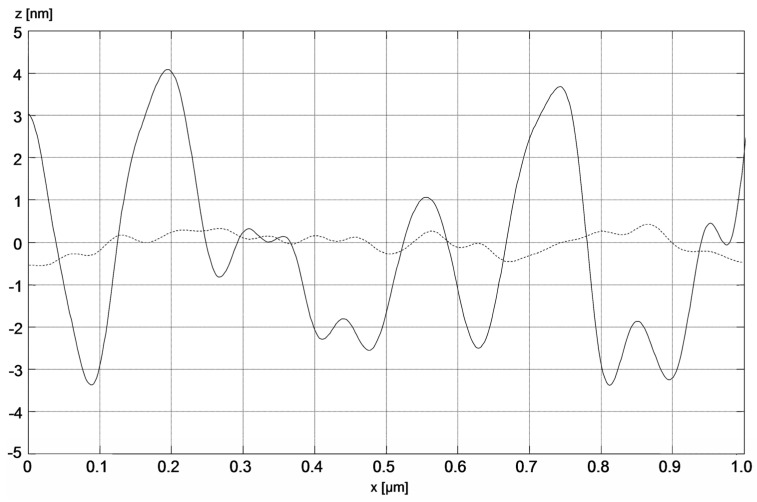
Section profiles of bimetallic Ag–Au (solid line) and single-metallic Au (dashed line) layers.

The image of the surface morphology of the bimetallic Ag-Au layer and the view of this layer’s cross-section, obtained by SEM, are presented in Figure 3a,b. Figure 3c presents the SEM image of a single Au layer. The results of AFM measurements of the Ag-Au bimetallic layer’s surface and the single Au layer’s surface are presented in Figure 4a,b. Representative profiles of cross-sections for these AFM measurement results are shown in Figure 5.

**Figure 6:**
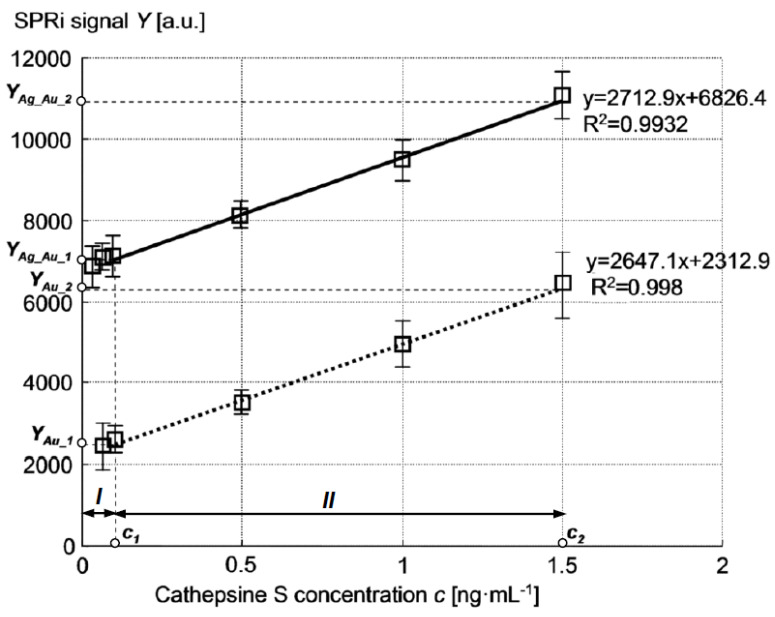
Calibration curves of cathepsin S SPRi Ag–Au (solid line) and Au (dotted line) biosensor.

A calibration curve for cathepsin S determination was plotted at previously established conditions: pH of 7.4 and antibody concentration of 20 ng·mL^−1^. The curve was determined in a range of various cathepsin S concentration values between 0.1 ng·mL^−1^ and 1.5 ng·mL^−1^. Graphs of calibration curves of biosensors made using Au and Ag–Au layers are shown in Figure 6. The figure presents only the linear range of the calibration curves for both glass slides. Both calibration curves show a similar slope but extremely different ranges of SPRi signal values. Precision and accuracy (represented by recovery) were tested for three different cathepsin S concentrations (depending on slide type): Au slide −0.1, 0.5, and 1.0 ng·mL^−1^, Ag–Au slide −0.05, 0.1, and 0.5 ng·mL^−1^. The number of measurements was 2 × 12 for each concentration. The results are shown in Table 1. Both slide types present an extremely low limit of detection (LOD) and limit of quantification (LOQ) values, but the Ag–Au slide shows better LOD and LOQ values. LOD was calculated as a sum of mean blank (matrix without analyte) and 3·SD of reagent blank. LOQ was calculated as a sum of mean blank and 10·SD of reagent blank (SD is the standard deviation).

## 4. Discussion

Bimetallic Ag–Au SPR systems with layer thicknesses similar to those applied in this study have been described in [30,43]. A high degree of consistency of results was obtained between the numerical model and experimental SPR results for the single- and bimetallic system of Au and Ag–Au layers (Figure 2). The details of the surface morphology of Ag–Au system of layers and its fracture, presented in Figure 3a,b, as well as the cross-sectional profile presented in Figure 5, indicate that the constant thickness values of individual layers adopted in modeling should be treated as mean values. Fine, convex, oval-shaped forms with transverse linear dimensions on the order of 10 to several dozen nanometers are dominant in the surface structure. An image of structures of a similar nature can be seen on the surface of the single Au layer (Figure 3c); however, they are finer, and the surface is smoother. General conclusions from SEM observations are confirmed by the results of AFM measurements (Figure 4a,b). RMS roughness values determined for the profiles from Figure 5 amounted to 2.12 nm and 0.15 nm, respectively, for Ag–Au and Au layers.

A characteristic feature arising from the comparison of both sensors’ calibration curves (Figure 6) is the similar level of Au and Ag–Au biosensors’ sensitivity in the linear range, indicated by the similar slope of the curves, where the signal of the Ag–Au sensor is at a level several times greater. Immobilization of cathepsin S receptors was performed identically on the Au surface of both sensor types; hence, the chemical characteristics of the sensing surface should be very similar in both sensors. The presence of differences between the calibration curves of the biosensors should therefore be ascribed to other types of characteristics of the Au and Ag–Au metallic layers.

The high goodness of fit of SPR curve modeling results to experimental results of reflectance measurement of the Ag–Au layer (Figure 2) suggests the possibility of using the SPR model to estimate sensitivity S_RI_—the first component of sensitivity S_ic_ from Equation (2) for the bimetallic layer, with the application of Winspall software. The good fit of the model’s results to the experiment indicates that the relative complex electric permittivity ε_Ag_ and ε_Au_ values used in the model account for losses present under the conditions of the conducted SPR measurement. These losses result from, among other things, the level of roughness of the metallic surface [44]. Hence, for a surface with a greater roughness, the value of the imaginary component ε_Ag_″ of the electric permittivity of Ag is slightly higher than that found in the literature for smoother silver surfaces [45]. It also seems that the Au sensor can be modeled in a similar fashion, and the results can be used for the analysis of its sensitivity.

**Figure 7:**
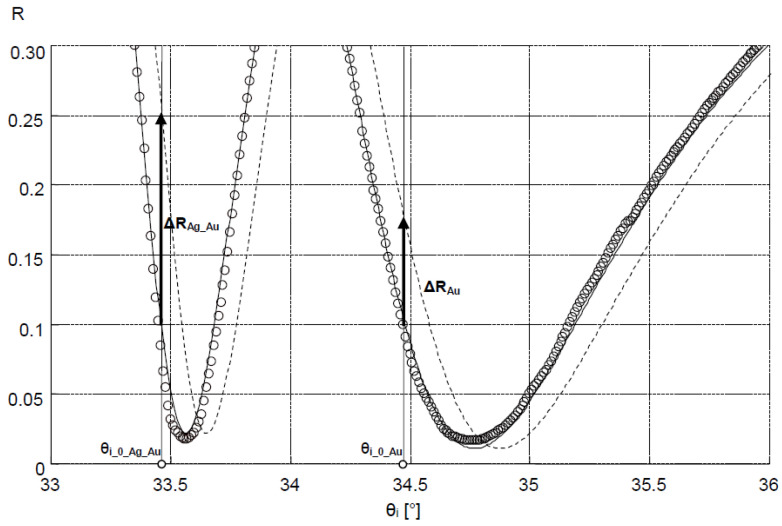
Changes in the position of the SPR model curves of Ag–Au and Au layers as a result of the increase in refractive index Δn and the related changes in reflectance ΔR_Ag_Au_ and ΔR_Au_ (dashed line: SPR curves corresponding to Δn increase); circles: experimental results to which SPR Ag–Au and Au model curves were fitted.

Sensitivity S_RI_ (for both Ag–Au and Au layers) was defined as the value of the quotient ΔY/Δn within the range of small values of increment Δn Equation (2). The output signal Y = A·ΔR was adopted as proportional to the increment of reflectance ΔR, determined for a constant value of angle θ_i___0_, selected in the linear part of the SPR curve with a large slope, so that angle θ_i___0_ corresponded to the reflectance value R = 0.1 at ΔR = 0 (Figure 7). The constant factor A was introduced in order to account for the 16-bit representation of the reading result (expressed in a.u.) indicated by the detector of the Kretschmann measuring system during the plotting of calibration curves (Figure 6). The value Δn = 0.0025 was adopted so that the ΔR value corresponded to the maximum change of reflectance of the cathepsin S biosensor. It was assumed that changes of refractive index n occur in the near-surface layer of the medium with relative permittivity ε_3_, adjoining the metallic layer (Figure 1). The thickness of this medium’s layer, equal to 100 nm, was accepted as the typical distance from the metal–dielectric boundary, on which the evanescent electromagnetic field has a relatively high intensity [43]. Figure 7 provides a graphical representation of position changes of the minimums of SPR curves plotted for Ag–Au and Au layers as a result of the increase of the refractive index by Δn, resulting in reflectance changes ΔR_Ag_Au_ and ΔR_Au_. Based on simulations conducted for Δn = 0.0025, reflectance increments of ΔR_Ag_Au_ = 0.153 and ΔR_Au_ = 0.072 were obtained (Figure 7). Using the general relationship S_RI_ = A·ΔR/Δn, described above, the value of S_RI_Ag_Au_ = A·61.2 RIU^−1^ was determined for the Ag–Au layer and S_RI_Au_ = A·28.8 RIU^−1^ for the Au layer. These components of sensitivity accept, in approximation, a constant value (S_RI_Ag_Au_ = const and S_RI_Au_ = const) within the range of refractive index changes Δn from 0 to 0.0025, and hence, within the entire range of changes of analyte concentration c, with which refractive index n is related by function n(c). The ratio of sensitivities takes the value S_RI_Ag_Au_/S_RI_Au_ = 2.1. 

Using the determined S_RI_Ag_Au_, S_RI_Au_ values, and biosensor calibration curves (Figure 6), it is possible to draw conclusions as to the mutual relationships between sensitivities S_nc_Ag_Au_ = ∂n_Ag_Au_/∂c and S_nc_Au_ = ∂n_Au_/∂c for sensors of both types. Using Equation (2) and the determinations given in Figure 6, the following dependencies can be written for concentration c within the range from 0 to c_1_ (analyte concentration interval I in Figure 6):

(3) $$Y_{Ag\_Au\_1}=\int_{0}^{c1}S_{RI\_Ag\_Au}{\left(\frac{\partial n_{Ag\_Au}}{\partial c}\right)}_{I}dc$$

(4) $$Y_{Au\_1}=\int_{0}^{c1}S_{RI\_Au}{\left(\frac{\partial n_{Au}}{\partial c}\right)}_{I}dc$$

where the expressions in parentheses denote sensitivities S_nc_i_ = ∂n_i_/∂c, in the general case of a non-linear characteristic, of individual biosensors within interval I. Substituting the non-linear characteristics S_nc_i_ of Ag–Au and Au sensors within interval I with their average, constant values ${\bar{S}}_{nc\_i}$, it can be written, based on (3) and (4), that:

(5) $$Y_{Ag\_Au\_1}=S_{RI\_Ag\_Au}{\bar{S}}_{{{}_{nc\_Ag\_Au\_}}_{I}}c_{1}$$

(6) $$Y_{Au\_1}=S_{RI\_Au}{\bar{S}}_{{}_{\begin{array}{l} nc\_Au\_I \end{array}}}c_{1}$$

Using (5) and (6) as well as the previously determined values of S_RI_Ag_Au_, S_RI_Au_, and the numerical values from Figure 6, it is possible to determine the mutual, quantitative relationship between the average sensitivities ${\bar{S}}_{nc\_i}$ of both types of sensors within interval I:

(7) $$\frac{{\bar{S}}_{{}_{nc\_Ag\_Au\_I}}}{{\bar{S}}_{{}_{nc\_Au\_I}}}=\frac{Y_{Ag\_Au\_1}}{Y_{Au\_1}}\frac{S_{RI\_Au}}{S_{RI\_Ag\_Au}}=1.4$$

Dependencies within the interval from c_1_ do c_2_ (analyte concentration c interval II in Figure 6) can be written analogously:

(8) $$Y_{Ag\_Au\_2}=Y_{Ag\_Au\_1}+\int_{c1}^{c2}S_{RI\_Ag\_Au}{\left(\frac{\partial n_{Ag\_Au}}{\partial c}\right)}_{II}dc$$

(9) $$Y_{Au\_2}=Y_{Au\_1}+\int_{c1}^{c2}S_{RI\_Au}{\left(\frac{\partial n_{Au}}{\partial c}\right)}_{II}dc$$

Adopting approximations analogously to those adopted in interval I, we obtain, in interval II:

(10) $$\frac{{\bar{S}}_{{}_{nc\_Ag\_Au\_II}}}{{\bar{S}}_{{}_{nc\_Au\_II}}}=\frac{\left(Y_{Ag\_Au\_2}-Y_{Ag\_Au\_1}\right)}{\left(Y_{Au\_2}-Y_{Au\_1}\right)}\frac{S_{RI\_Au}}{S_{RI\_Ag\_Au}}=0.5$$

The results obtained indicate that, for very low analyte concentrations (interval I), the mean sensitivity ${\bar{S}}_{nc}$ of the Ag–Au sensor is nearly half higher than the sensitivity of the Au sensor Equation (7). In turn, for concentration c corresponding to the linear range of both biosensors’ calibration curves (interval II), sensitivity ${\bar{S}}_{nc}$ of the Ag–Au sensor is on the order of half of the sensitivity of the Au sensor Equation (10).

Preparation and immobilization of bioreceptors were realized in the same manner on the Au surfaces of both sensors. Nevertheless, as demonstrated above, the sensitivities S_nc_i_ of both sensors differ. It is important to explain the reasons for the above-described differences. The factor fundamentally influencing the value of sensitivity S_nc_ is, in the general case, the efficiency of chemical interaction of the sensing surface (with deposited bioreceptors) with analyte molecules. Higher efficiency is expressed by a greater increment of the number of analyte-bioreceptor pairs, and in effect, by a greater change Δn of refractive index n at a specific level of changes of concentration c of analyte molecules in the layer of the solution coming into contact with the sensing surface. 

**Figure 8:**
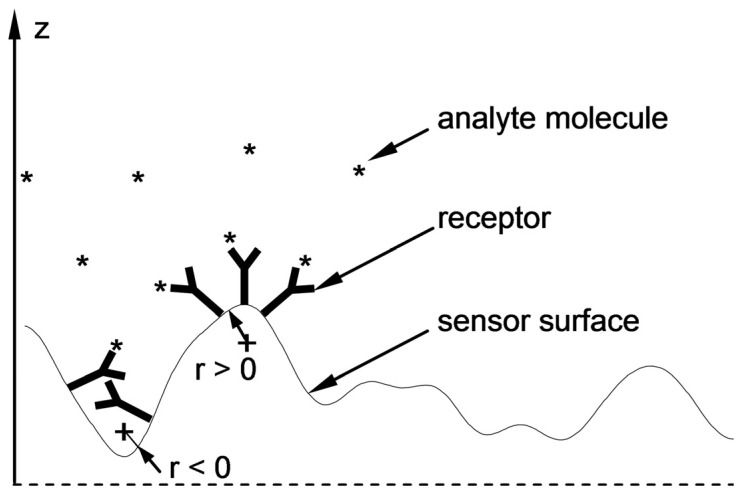
Local “expansion” of the surface with a positive radius of curvature (r > 0) and the related geometric arrangement of the receptors that facilitate the attachment of analyte molecules (right side) and unfavorable geometric conditions (r < 0) for bioreceptor–analyte pair formation (left side).

In search of the causes of these differences and comparing the cross-sectional profiles of the surfaces in Figure 5, one can perceive distinct differences in the geometric characteristics of both sensors’ surfaces. Surface roughness parameters are usually linked to sensor performance [44]. As shown above, the influence of roughness of the Ag–Au layer was already accounted for in the component of sensitivity S_RI_ through a slightly higher value of the imaginary component of relative electric permittivity of Ag. It seems that S_nc_ sensitivity can be linked to another aspect of the geometric characteristics of the sensing surface. It seems that convex fragments of the surface (Figure 8) with a positive sign of radius of curvature r and those more deeply immersed in the tested solution, with the bioreceptors located on the surface, will more intensively bond with analyte molecules in comparison to concave fragments of the surface in depressions. The local “expansion” of the convex surface fragments and the geometric arrangement of receptors associated with it should facilitate bonding with analyte molecules. In addition, the diffusive nature of transport of these molecules, occurring in the direction of negative values of the z coordinate, fosters adsorption of the analyte at the highest points on the surface, where the concentration of analyte molecules available for reaction is the greatest during the active phase of the biosensor’s operation. Therefore, these fragments of the surface should be characterized by a higher increment value of refractive index n, arising from the greater increment of the density of adsorbed analyte molecules as concentration c increases, and thus, by a greater value of sensitivity S_nc_ than the sensitivity of concave areas (r < 0) with a lower z coordinate.

**Figure 9:**
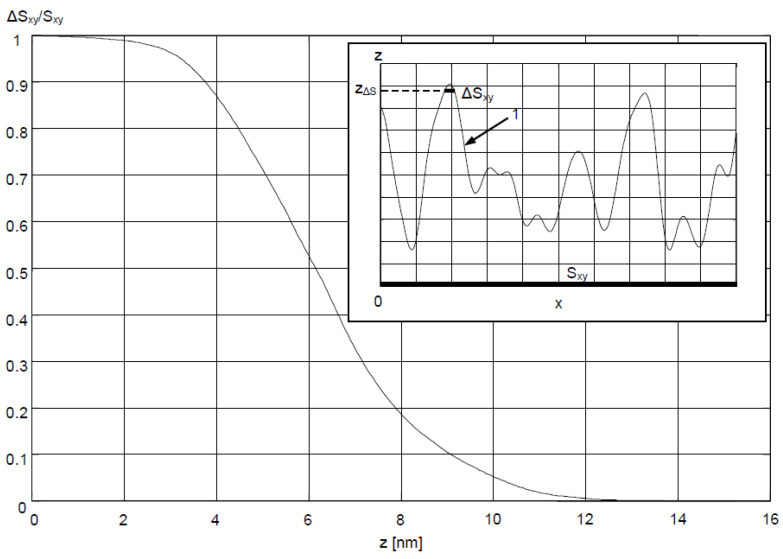
ΔS_xy_/S_xy_ as a function of z coordinate for Ag–Au sensor surface; upper right corner: side view of ΔS_xy_ corresponding to sensor surface 1 located above coordinate z_ΔS_ and the projection of the area S_xy_ of the sensor surface onto the xy plane.

In order to account for the geometric features of Ag–Au sensor surface described above, a description of the surface was proposed, involving the determination of the ratio ΔS_xy_/S_xy_ of the projection of the surface ΔS_xy_ above the z_ΔS_ coordinate to the projection of area S_xy_ of the entire sensing surface (Figure 9) onto the xy plane. The results of AFM measurements (Figure 4a) were used for calculations. It was accepted that the z coordinate is equal to 0 for the lowest point on the surface. Moving from the highest peak on the surface z_max_ in the direction of decreasing z values, increment ΔS_xy_ first encompasses the areas most deeply immersed in the analyte, mostly having a positive sign of radius of curvature r (which can be concluded from Figure 5), and therefore, with a postulated high value of S_nc_. Graph of ΔS_xy_/S_xy_(z) in Figure 9, shows that for the Ag–Au sensor, the share ΔS_xy_/S_xy_ of fragments of the surface with a high z coordinate (and therefore high S_nc_ value) is very low. Thus, as the analyte’s concentration grows, starting from the value of c = 0 in analyte concentration interval I, bioreceptor–analyte pairs will be formed with the greatest intensity in relatively few sites with a high z coordinate and S_nc_ value Equation (7). As concentration c reaches values in interval II, these sites will quickly be depleted in terms of reactive capacity. Next, the remaining, substantially larger sensing surface, characterized by lower values of z coordinate and sensitivity S_nc_, will take part in the adsorption process. For the significantly smoother surface of the Au sensor, it should be expected that the mean S_nc_ will be greater than for the Ag–Au sensor within the linear range in interval II Equation (10). 

It can be observed that the presented description corresponds to the characteristics of both sensors’ calibration curves. The higher level of the Ag–Au sensor signal results from the higher values of both sensitivity components (S_RI_ and S_nc_) in relation to the Au sensor in analyte concentration interval I Equations (5) and (6). In interval II, the products S_RI_·S_nc_ of both sensors have similar values, so the calibration curves have a similar slope despite the different signal levels. 

At the same time, the higher signal values of the Ag–Au sensor result in a lower noise level of this sensor at the same intensity of the measuring beam during the operation of both types of sensors. Therefore, the Ag–Au sensor should be characterized by greater measuring resolution and a lower limit of detection value in comparison with the Au sensor. A comparison of the LOD and LOQ values of both sensors (Table 1) confirms these dependencies. 

## 5. Conclusions

It follows from the above discussion that increasing the value of the ΔS_xy_/S_xy_ expression for convex areas of the sensory surface with a high z coordinate should result in an increase in the S_nc_ sensitivity component, and thus the overall sensitivity S_ic_ of the sensor for low analyte concentrations. The shape of the sensory surface should then take the form of, for example, a dense matrix of convex vertices with the same high z coordinate. Such a proposal is a new idea. Controlled preparation of surface relief structures with such characteristics is a potentially difficult task. The actions taken so far in the design of sensors are aimed rather at obtaining the smoothest sensory surface possible. Testing this hypothesis could be a future research task. It is worth noting that the advantages of surfaces with a higher roughness level have been demonstrated, for example, when optimizing the surface chemistry of impedimetric biosensors [46]. Nevertheless, the issues related to the greater degree of complexity of the production of bimetallic structures, affecting their production costs, the potentially higher susceptibility to oxidation of the Ag–Au bimetallic sensory layer, and the related storage requirements, require further research in order to optimize their properties. The presented hypothesis does not cover other factors that could have a potential influence on the characteristics of calibration curves, e.g., the effect of position change of the SPR curve’s minimum caused by the dissolution of certain ingredients present in the sensing zone in the buffer. Determination of the occurrence of these additional effects requires further research.

## Figures and Tables

**Table 1:** Precision and accuracy for cathepsin S determination (n = 24) with the use of different slide types.

**Glass slide/Au**				
***c*_added_** **ng·mL^−1^**	***c*_found_** **ng·mL^−1^**	**SD** **ng·mL^−1^**	**Recovery** **%**	**RSD** **%**
0.1	0.113	0.0387	113.4	34.1
0.5	0.529	0.0449	105.9	8.5
1.0	0.981	0.0852	98.1	8.7
LOD = 0.034 ng·mL^−1^ LOQ = 0.113 ng·mL^−1^				
**Glass slide/Ag–Au**				
***c*_added_** **ng·mL^−1^**	***c*_found_** **ng·mL^−1^**	**SD** **ng·mL^−1^**	**Recovery** **%**	**RSD** **%**
0.05	0.052	0.0076	104.3	14.5
0.1	0.097	0.0088	97.1	9.1
0.5	0.507	0.0592	101.5	11.7
LOD = 0.031 ng·mL^−1^ LOQ = 0.093 ng·mL^−1^				

## Data Availability

Not applicable.
